# Complete Genome Sequences of *Rhodoluna* sp. Strains KAS3 and KACHI23, Isolated from Lake and River Surface Water

**DOI:** 10.1128/mra.01122-22

**Published:** 2022-11-29

**Authors:** Yusuke Ogata, Keiji Watanabe, Shusuke Takemine, Kaoru Kaida, Maki Tanokura, Wataru Suda

**Affiliations:** a RIKEN Center for Integrative Medical Sciences, Yokohama, Kanagawa, Japan; b Center for Environmental Science in Saitama, Kazo, Saitama, Japan; University of Delaware

## Abstract

The genus *Rhodoluna* belongs to the ubiquitous freshwater bacterioplankton tribe Luna1-A2. Here, we report the complete sequences of *Rhodoluna* sp. strains KAS3 and KACHI23, which were isolated from freshwater lake and river surface water in Japan.

## ANNOUNCEMENT

The bacterioplankton of the genus *Rhodoluna*, which belongs to the Luna1-A2 tribe, have worldwide distribution, with sometimes relatively high abundance in the sequence databases (1, 2). To date, only two species (Rhodoluna lacicola and Rhodoluna limnophila) have been described (3, 4). In comparison with its ubiquity, the genomic information on *Rhodoluna* is very limited. Therefore, accessing genomic information for *Rhodoluna* bacteria is important for providing physiochemical and ecological insights into the genus.

Here, we report the complete genome sequences of two *Rhodoluna* sp. strains, KAS3 (JCM 18064) and KACHI23 (JCM 32204), which were isolated from a shallow eutrophic lake surface water sample collected from Lake Kasumigaura, Tsuchiura, Ibaraki, Japan (36°02′06″N, 140°24′30″E), on 10 November 2010 and a river surface water sample collected from the Ichino River (Kachi Bridge), Yoshimi, Saitama, Japan (36°01′08.8″N, 139°28′23.9″E), on 2 July 2013, respectively. The samples were filtered through a disposable syringe equipped with a 0.7-μm particle-retention glass-fiber filter (Puradisc 25 GF/F disposable filter device; Whatman, Springfield Mill, UK). After filtration, the filtrates were spread on modified Reasoner’s 2A (MR2A) agar plates and incubated at 25°C for 2 to 5 days (5). A single bacterial colony for each strain was picked, inoculated into sterilized MR2A liquid medium (pH 7.2), and incubated at 25°C for 2 days with reciprocal shaking (120 rpm), and the pure strain cell suspensions were preserved at −80°C as stocks in MR2A broth supplemented with 20% (wt/vol) glycerol. Cells from the glycerol stocks were inoculated and cultured in MR2A liquid medium, harvested by centrifugation, and used for genomic DNA extraction.

Genomic DNA was extracted from strains KAS3 and KACHI24 using enzymatic lysis and phenol-chloroform-isoamyl alcohol extraction as described previously (6). Whole-genome sequencing was performed using a Sequel II system (Pacific Biosciences of California, Inc. [PacBio], Menlo Park, CA, USA). The library was prepared with the SMRTbell Express template preparation kit v2.0 (PacBio) with DNA shearing by g-TUBE (Covaris, Woburn, MA, USA) and with a target length of 10 to 15 kb. The PacBio reads were converted to circular consensus sequencing (CCS) reads using CCS software v6.2.0 (https://github.com/PacificBiosciences/ccs) and assembled using the assembler Canu v2.1.1 with specified parameters minReadLength=2200 and minOverlapLength=2200 (7), and the generated contig was checked for circularization to remap the CCS reads by Minimap2 v2.24-r1122 (8). The genomic sequence obtained was annotated and rotated to start at *dnaA* using DFAST (https://dfast.nig.ac.jp) (9). Default parameters were used for all software analysis unless otherwise specified. The reads obtained and genomic sequences generated are summarized in Table 1. The average nucleotide identity by orthology (OrthoANI) and average amino acid identity (AAI) between strain KAS3 and R. limnophila 27D-LEPI^T^ were 93.66% and 97.00%, respectively, and those between strain KACHI23 and R. lacicola MWH-Ta8^T^ were 97.27% and 97.44%, respectively. Thus, strains KAS3 and KACHI23 belong to the genus *Rhodoluna*.

**TABLE 1:** Summarized data on reads and contigs obtained for *Rhodoluna* sp. strains KAS3 and KACHI23

Parameter	Data for strain:	
	KAS3	KACHI23
No. of quality-passed Sequel reads	30,000	30,000
Total size of quality-passed Sequel reads (bp)	494,698,795	416,995,817
*N*_50_ of quality-passed Sequel reads (bp)	16,580	13,774
Total no. of contigs	1	1
BioProject accession no.	PRJDB14318	PRJDB14318
BioSample accession no.	SAMD00533725	SAMD00533726
SRA accession no.	DRR410665	DRR410666
Genome size (bp)	1,338,021	1,408,920
GC content (%)	53.8	51.5
GenBank/ENA/DDBJ accession no.	AP026910	AP026911

Based on annotation results for the KAS3 and KACHI23 genome sequences, a gene predicted to encode polyphosphate kinase (*ppk*), which is associated with intracellular accumulation of polyphosphate, was identified, in addition to a gene encoding a putative xanthorhodopsin-like protein associated with light-driven, proton-pumping rhodopsin. Furthermore, these strains also contained genes encoding putative alanine dehydrogenase-like proteins associated with ammonification.

### Data availability.

The chromosome sequences and reads for strains KAS3 and KACHI23 were deposited in the GenBank/ENA/DDBJ database under the accession no. AP026910 and AP026911, which are linked to the BioProject accession no. PRJDB14318, BioSample accession no. SAMD00533725 and SAMD00533726, and DDBJ Sequence Read Archive accession no. DRR410665 and DRR410666, respectively. The details are summarized in Table 1.
